# Intravenous nitroglycerin does not preserve gastric microcirculation during gastric tube reconstruction: a randomized controlled trial

**DOI:** 10.1186/cc5043

**Published:** 2006-09-13

**Authors:** Marc Buise, Jasper van Bommel, Alexander Jahn, Khe Tran, Huug Tilanus, Diederik Gommers

**Affiliations:** 1 Department of Anesthesiology, Erasmus MC Rotterdam, Gravendijkwal, 3015 CE Rotterdam, The Netherlands; 2 Department of Intensive Care, Erasmus MC Rotterdam, Gravendijkwal, 3015 CE Rotterdam, The Netherlands; 3 Department of Surgery, Erasmus MC Rotterdam, Gravendijkwal, 3015 CE Rotterdam, The Netherlands

## Abstract

**Introduction:**

Complications of oesophagectomy and gastric tube reconstruction include leakage and stenosis, which may be due to compromised microvascular blood flow (MBF) in gastric tissue. We recently demonstrated that decreased MBF could be improved perioperatively by topical administration of nitroglycerin. The aim of the present study was to investigate whether nitroglycerin, administered intravenously during gastric tube reconstruction, could preserve tissue blood flow and oxygenation in the gastric fundus, and reduce the incidence of postoperative leakage.

**Methods:**

In this single-centre, prospective, double-blinded study, we randomized 32 patients scheduled for oesophagectomy into two groups. The intervention group received intravenous nitroglycerin during gastric tube reconstruction, and the control group received normal saline. Baseline values for MBF, microvascular haemoglobin oxygen saturation and microvascular haemoglobin concentration were determined at the gastric fundus before and after gastric tube construction and after pulling up the gastric tube to the neck.

**Results:**

MBF and microvascular haemoglobin oxygen saturation decreased similarly in both groups during gastric tube reconstruction and were comparable. The oesophageal anastomosis was controlled by contrast radiography before discharge from the hospital; leakage was observed in two patients (13%) in the nitroglycerin group and five patients (31 %) in the control group (not significant).

**Conclusion:**

Under stable systemic haemodynamic conditions, continuous intravenous administration of nitroglycerin could not prevent deterioration in gastric microvascular perfusion and microvascular haemoglobin saturation during gastric tube reconstruction. (Trial registration number NCT 00335010.)

## Introduction

Oesophagectomy with direct reconstruction of the digestive tract remains the most successful therapy for oesophageal cancer. Frequent complications of the gastro-oesophageal anastomosis include leakage (5–26%) and anastomotic stenosis (12–40%), which have been attributed to decreased microvascular blood flow (MBF) and concomitant compromised oxygenation of the gastric tube [1,2]

The decrease in MBF is accounted for predominantly by diminished arterial supply of the gastric tube caused by ligation of several gastric arteries during the course of the procedure. On the other hand, venous congestion has been proposed to contribute to this reduced MBF as well [3]. We recently demonstrated a decrease in MBF but not in microvascular haemoglobin oxygen saturation (μHbSO_2_) during gastric tube reconstruction, using simultaneous measurement of MBF and μHbSO_2 _[4]. We showed that the decreased MBF could be improved with topical administration of nitroglycerin.

This effect of nitroglycerin, which must be considered temporary because of the short half-life of nitroglycerin, depends entirely on the accessibility of gastric tube tissue. Therefore, we considered this observation proof of principle, and hypothesized that it should be possible to establish a similar effect with intravenous administration of nitroglycerin. If so, then the effect could easily be prolonged during the days following surgery. Our hypothesis was supported by data reported by other investigators, who were able to improve tissue perfusion in septic patients [5] or during cardiopulmonary bypass [6] with intravenous administration of nitroglycerin. Based on these observations, we hypothesized that systemic administration of nitroglycerin can preserve gastric MBF and μHbSO_2 _during gastric tube reconstruction.

## Materials and methods

With the approval of the local institutional human investigation committee, and after obtaining written informed consent, 32 patients were included between May 2004 and March 2005. All patients were in physical status I and II, according to the American Society of Anesthesiologists classification [7].

General anaesthesia was induced with propofol (1–2 mg/kg), sufentanil (0.1–0.3 μg/kg) and rocuroniumbromide (0.6 mg/kg). Anaesthesia was maintained with isoflurane (0.8–1.2 end-tidal %) in all patients. Before induction of general anaesthesia, a mid-thoracic epidural catheter was placed (between Th6 and Th8) to provide peroperative and postoperative analgesia. In all patients epidural blockade was started with a bolus of 10 ml bupivacaine 0.25%, before starting the operation. After 90 min, a mixture of bupivacaine 0.125% with fentanyl 2.5 μg/ml was administered through the epidural catheter at a dosage of 10 ml/hour.

All patients were mechanically ventilated to achieve an arterial carbon dioxide tension of 4.5–5.5 kPa. Fractional inspired oxygen was initially set at 40%, but this was increased to 70% before fashioning the gastric tube. Positive end-expiratory pressure was set at 5 cmH_2_O. In all patients standard haemodynamic monitoring was used, including radial arterial blood pressure and right atrial pressure (RAP) measurements. Fluid management was performed using crystalloids and hydroxyethyl starch (Voluven^®^; Fresenius Kabi, 's-Hertogenbosch, The Netherlands) in order to maintain mean arterial pressure above 60 mmHg and RAP above 10 mmHg. The attending anaesthesiologists were advised to use vasopressors when the volume of colloids exceeded 2 l; the agent of choice was phenylephrin. Arterial and central venous oxygen and carbon dioxide partial pressures, haemoglobin concentration and haemoglobin saturation were determined (ABL 707; Radiometer, Copenhagen, Denmark).

### Operation technique

Two operation techniques were used: transhiatal and transthoracic oesophagectomy. Although these approaches differ, in both techniques the gastric tube is constructed in a similar manner by ligation of the left gastric artery, the right gastric artery, the short gastric arteries and the left gastroepiploic artery, and it is then fashioned along the greater curvature. As a consequence, the arterial supply of the upper part of the gastric tube depends exclusively on the right gastroepiploic arterial arcade. After transecting the oesophagus in the neck and stripping of the oesophagus, the gastric tube was pulled up via the prevertebral route where an oesophagogastrostomy was created. In all patients an end-to-side anastomosis was hand sewn with a single layer using absorbable monofilament suture (PDS 3–0, Ethicon; Johnson & Johnson, Amersfoort, The Netherlands).

All operations were performed by the same surgical team (KT and HT).

### Reflection spectrophotometry and laser Doppler flowmetry

The microvascular parameters MBF, μHbSO_2 _and microvascular haemoglobin concentration (μHbcon) were determined simultaneously using the O2C^® ^(Lea Medizin Technik, Giesen, Germany). This device combines two optical techniques, namely laser Doppler flowmetry (LDF) and reflectance spectrophotometry (RS), in one optic fibre. There is no interference between the two techniques because they operate at different light wave ranges. In the present study a flat probe was used, with a measurement depth of 4–6 mm.

μHbSO_2 _was measured using RS. The tissue is illuminated with visible white light (500–630 nm), which is backscattered mainly by mitochondria and changed in colour by haemoglobin according to its oxygen saturation status. This reflected spectrum is detected and analyzed by a spectrophotometer with a frequency of more than 100 Hz; a mean of these values is provided every 2 s. In addition, the μHbcon is calculated as a relative value in arbitrary units (AU). The clinical usability of RS and its value for the assessment of microvascular oxygenation were demonstrated previously [8].

LDF is a well established technique for the assessment of microvascular perfusion, and has been used frequently during gastric tube reconstruction [3,4,9]. Using LDF, MBF is determined by analysis of the power spectra from moving blood cells, generated by Doppler frequencies of backscattered laser light (820 nm). The MBF value is defined mathematically as the first moment of the Doppler power spectra, so it relates to the velocity of the erythrocytes multipled by the number of moving erythrocytes, and it is described in AU. Changes in MBF have been qualitatively related to the occurrence of intestinal tissue ischaemia. For instance, Pierie and coworkers [9] demonstrated that a reduction in gastric MBF of more than 70% from baseline values was a predictor of impaired healing of the cervical oesophagogastrostomy. Similarly, during intestinal hypotension a comparable decrease in jejunal mucosal perfusion was associated with increased lactate production [10].

### Study protocol

Patients were randomized into two groups of 16 patients each. Randomization was performed by drawing of closed envelopes. The treatment group received intravenous nitroglycerin (Nitro Pohl; Transmedico BV, Weesp, The Netherlands) at a dosage of 1 μg/kg per min, started immediately following induction of anaesthesia. The control group received intravenous NaCl 0.9% at a similar infusion rate, started at the same time. The researcher and the attending anaesthesiologist were blinded as to the content of the syringe.

After opening the abdomen, but before compromising the vascularization of the stomach, baseline values (T0) of MBF, μHbSO_2 _and μHbcon were collected. An average of measurements over 1 min (30 values) was obtained from two gastric areas; the pre-pyloric antrum and the fundus of the stomach where the future anastomosis of the gastric tube was expected. The probe was placed by the surgeon, gently touching the surface of the serosal side of the stomach. Pressure artifacts were identified by an obvious decrease in signal in both LDF and RS curves, and a change in configuration of the RS signal. After T0 the measurements were repeated two further times: after construction of the gastric tube (T1) and after pulling up the gastric tube to the neck (T2). Arterial and central venous blood gas analysis was performed simultaneously. At the end of the operation the study medication was stopped.

As part of clinical practice following gastric tube reconstruction, all patients underwent a contrast radiography examination of the oesophagogastrostomy after at least 7 days or before leaving the hospital.

### Statistical analyses

In our previous study [4], in which patient served as their own controls, we found a increase in MBF from 52 AU to 100 AU after application of nitroglycerin, with a standard deviation of 34.8 and 53.4, respectively [4]. A sample size of 16 in each group has 80% power to detect a difference between means of 45 with a significance level (α) of 0.05 (two-tailed).

Values are reported as mean ± standard error. Each variable was analyzed using analysis of variance for repeated measurements. When appropriate, *post hoc *analyses were performed using the Bonferoni test. Differences between treatment and control groups at each time point were analyzed using an unpaired *t*-test. Incidences of postoperative leakage in the groups were compared using a Fischer test. *P *< 0.05 was considered statistically significant. All analyses were performed using Graphpad Prism (version 3.0; Graphpad Software, San Diego, CA, USA).

## Results

Demographic and operation characteristics are summarized in Table 1. In the nitroglycerin group 15 people were operated on using the transhiatal approach as compared with 12 patients in the control group. There was a significant difference in fluid volume administration: 6.5 ± 1.3 l in the nitroglycerin group versus 7.7 ± 1.7 l in the control group. Total perioperative blood loss was similar in the two groups. Both groups received equal amounts of vasoactive medication. Mean arterial blood pressure was comparable throughout the procedure and did not change significantly in either group. Heart rate was higher in the nitroglycerin group during the entire operation than in the control group. RAP was significantly higher in the control group than in the nitroglycerin group at baseline, and decreased compared with baseline at T2 (Table 2).

There was a significant difference in the central venous oxygen saturation between the control and the nitroglycerin groups at baseline. The arterial oxygen tension values were similar between groups throughout the procedure. There were significant differences in arterial haemoglobin concentration between baseline and subsequent time points in the two groups, and there was a difference in haemoglobin concentration between groups at T1 (Table 3).

With respect to microvascular parameters, baseline values were similar in the two groups for all parameters at the gastric fundus and pylorus. There was no significant change in or difference between the two groups in MBF, μHbSO_2 _and μHbcon at the pyloric part of the stomach during the procedure (data not shown).

**Figure 1:**
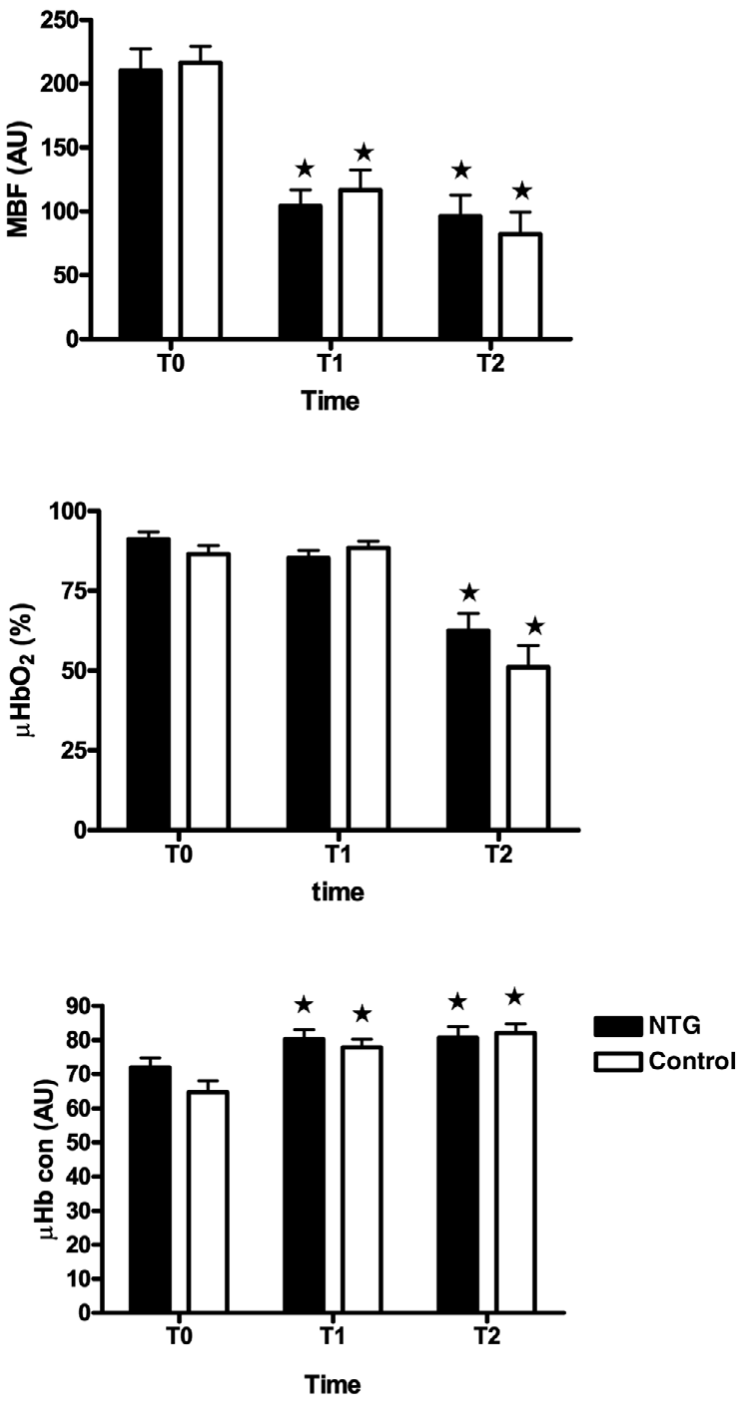
Microvascular parameters. MBF, μHbSO_2 _and μHbcon measured at the fundus of the stomach during gastric tube reconstruction. The MBF decreased between T0 and T1 and the μHbSO_2 _decreased between T1 and T2. Values are expressed as mean ± standard error. *P < 0.05 versus baseline (T0). μHbcon, microvascular haemoglobin concentration; μHbSO_2_, microvascular haemoglobin oxygen saturation; MBF, microvascular blood flow; T0, baseline; T1, after construction of the gastric tube; T2, after pulling up the gastric tube to the neck.

As can be seen in Figure 1, fundus MBF was 210 ± 17 AU in the nitroglycerin group and 216 ± 13 AU in the control group at baseline (not significant). There was a decrease at T1 in both the nitroglycerin group and the control group, but there was no further decrease from T1 to T2 in the two groups. μHbSO_2 _at T0 was 91 ± 2% in the nitroglycerin group and 86 ± 3% in the control group (not significant). There was no significant change in μHbSO_2 _in the two groups between T0 and T1. At T2, however, μHbSO_2 _decreased significantly in both groups, to 63 ± 5% in the nitroglycerin and 51 ± 7% in the control group. The μHbcon increased significantly between baseline and T1, from 72 ± 3 AU to 80 ± 3 AU in the nitroglycerin group and from 65 ± 3 AU to 78 ± 3 AU in the control group. There was no difference between T1 and T2.

All patients underwent a contrast radiography examination of the oesophagogastrostomy. Two cases of anastomotic leakage occurred in the nitroglycerin group (12%) and five occurred in the control group (31%); this difference was not statistically significant (*P *= 0.19). We did not differentiate between minor or major, clinically relevant leakage.

## Discussion

In the present study we were unable to prevent a decrease in gastric fundus microvascular perfusion and oxygenation during gastric tube reconstruction with continuous intravenous administration of nitroglycerin. This substance acts as a nitric oxide (NO) donor; in the vascular endothelium NO functions as a regulator of vascular tone, and thereby of microvascular perfusion [11]. NO plays an important role in the autoregulation of gastric mucosal blood flow, and it is likely that NO plays a role in protecting the gastric mucosa and preserves mucosal integrity [12,13]. During conditions of decreased flow, use of nitroglycerin was shown to be effective in improving tissue perfusion [4-6].

Nevertheless, the present results are in contrast with the findings of our previous study [4], in which gastric MBF could be improved with application of nitroglycerin locally on the gastric tube tissue. It cannot be ruled out that the dose of nitroglycerin used in the present study might have been insufficient to establish effective tissue concentrations in the gastric tube. We opted for a dosage of 1 μg/kg per min, which was in the same range as that used in the studies of conducted by Spronk and coworkers [5] (0.5 mg bolus followed by 33.3 μg/min) and Iribe and colleagues [6] (0.5–2.0 μg/kg per min). In both studies tissue perfusion could be significantly improved with intravenous nitroglycerin.

With topical administration, tissue concentrations must have been relatively high. If we had aimed to establish similar tissue concentrations or used an improvement in MBF as a therapeutic end-point, then greater amounts of nitroglycerin might have been required. On the other hand, during gastric tube reconstruction perfusion pressure (arterial blood pressure) is considered critical for adequate tissue perfusion and thus healing of the proximal anastomosis. Therefore, we decided not to use higher concentrations of nitroglycerin in our study to prevent its systemic effects.

Haemodynamic stability is demonstrated by the unchanged mean arterial pressure and RAP during the entire study protocol in the nitroglycerin group. There was a difference in the central venous haemoglobin oxygen saturation between the nitroglycerin and NaCl groups at T0, but this was not related to a difference in microvascular saturation. We did not measure cardiac output because we believe that there is no relation between changes in cardiac output and changes in microvascular blood flow, as was recently demonstrated by De Backer and coworkers [14].

Although one might assume that systemic circulatory capacitance is increased by nitroglycerin, the experimental group received less fluid during the procedure. Several reasons can have contributed to this finding. One is that in this study hypotension was primarily treated with fluid instead of vasopressors. As a result, too much fluid was administered in both groups with regard to the end-points for fluid administration. Looking at RAP, it can be seen that in all patients RAP was well above 10 mmHg during the entire procedure. If we had adhered to these end-points more strictly, then less fluid might have been administered, certainly in the control group, and differences in venous capacitance between the two groups might have been more marked. In the experimental conditions employed, only at baseline was RAP lower in the nitroglycerin group.

Another reason is that we cannot rule out the possibility that the dosage of nitroglycerin was simply too low to cause an effect on systemic circulatory capacitance, and therefore did not lead to increased administration of fluids in the experimental group. Whether a higher dose of nitroglycerin in combination with more fluids would have an effect on the microcirculation remains speculative.

Despite the differences in fluid administration, haemoglobin concentrations in the two groups were in the same range throughout the study period. Based on the American Society of Anesthesiologists guidelines for blood transfusion, a permissive anaemia strategy was used. Acute anaemia in the absence of hypovolaemia is known to have an effect on tissue perfusion. Microvascular perfusion is augmented by an increase in the amount of perfused capillaries in the tissue (capillary recruitment) and by vasodilatation of microvessels already perfused [15-17]. As a result, the absolute amount of oxygen transported by the capillaries can be maintained. In the splanchnic tissues, NO is thought to play an important role in this process [18,19]. It can be hypothesized that, under these circumstances, administration of an NO donor such as nitroglycerin might not have as much an effect as when thre are higher haemoglobin levels. This mechanism might have interfered with the effect of nitroglycerin administration.

The simultaneous decrease in MBF and increase in μHbcon, followed by a decrease in μHbSO_2 _at a later stage, implies that venous congestion plays an important role in the decrease in gastric tissue perfusion during gastric tube reconstruction. This mechanism has been proposed by others as well [3]. Blood flow in the mucosal and serosal layers of the gut is known to behave differently under certain circumstances. In addition, the distinct effects of NO donors such as nitroglycerin on the various layers of the gastric tissue during this kind of surgery are unknown. For obvious reasons, we were only able to apply the O2C^® ^probe on the serosal side of the gastric tube. Because the measurement depth of this probe is in the 4–6 mm range, we cannot distinguish between the different layers of the gastric tissue. It is therefore very difficult to draw any conclusions regarding differential tissue blood flow changes in our study.

Finally, the incidence of anastomotic leakage is relatively high in the total study population (22%); this included clinically relevant leakage as well as leakage restricted to radiological signs only. Although not supported by the microvascular data, we observed a tendency toward a lower incidence of anastomotic leakage in the nitroglycerin group. This result did not achieve statistical significance, but the study was not designed for that purpose either. Larger patient numbers might be required to evaluate this.

## Conclusion

Intravenous administration of nitroglycerin at a dosage of 1 μg/kg per min does not prevent the decrease in gastric MBF and μHbSO_2 _that occur following gastric tube reconstruction. Further research is necessary to gain more insight into the effect of NO donors on impaired microvascular perfusion and oxygenation in general, and its relation to anastomotic complications following oesophagogastrostomy specifically.

## Key messages

• Venous congestion plays an important role in the impairment in microvascular perfusion that occurs following gastric tube reconstruction.

• Intravenous nitroglycerin at a rate of 1 μg/kg per min does not prevent the deterioration in gastric microcirculation during gastric tube reconstruction.

## Abbreviations

AU = arbitrary units; μHbcon = microvascular haemoglobin concentration; μHbSO_2 _= microvascular haemoglobin oxygen saturation; LDF = laser Doppler flowmetry; MBF = microvascular blood flow; NO = nitric oxide; RAP = right atrial pressure; RS = reflectance spectrophotometry.

## Competing interests

The authors declare that they have no competing interests.

## Authors' contributions

MB was the principal investigator. JVB acted as study monitor, assisted in the data analysis and helped to draft the manuscript. AJ performed data collection. KT and HT performed surgery and measurements, and participated in data interpretation. DG participated in the study design and coordination, and helped to draft the manuscript. All authors read and approved the final manuscript.

## Figures and Tables

**Table 1:** Demographic and operative characteristics

Characteristic	Group	
	Nitroglycerin	NaCl (control)
Male/female (*n*)	14/2	13/3
Age (years)	62 ± 11	61 ± 9
Operation time (min)	236 ± 52	245 ± 55
Infusion (l)	6.5 ± 1.3*	7.7 ± 1.7
Blood loss (l)	1.1 ± 0.1	1.4 ± 0.2

Values are expressed as mean ± standard error. *P < 0.05 NaCl (control) versus nitroglycerin.

**Table 2:** Haemodynamic parameters

Parameter	Group	Time point		
		
		T0	T1	T2
MAP (mmHg)	NTG	70 ± 2	72 ± 3	70 ± 2
	NaCl	75 ± 4	73 ± 3	72 ± 2
RAP (mmHg)	NTG	13 ± 1*	12 ± 1	11 ± 1
	NaCl	16 ± 1	14 ± 1	13 ± 1**
HR (beats/min)	NTG	86 ± 3*	83 ± 3*	88 ± 3*
	NaCl	74 ± 3	71 ± 3	75 ± 4

Values are expressed as mean ± standard error. *P < 0.05 NTG versus NaCl (control); **P < 0.05 versus T0 (baseline). HR, heart rate; MAP, mean arterial pressure; NaCl, normal saline (control); NTG, nitroglycerin; RAP, right atrial pressure; T0, baseline; T1, after construction of the gastric tube; T2, after pulling up the gastric tube to the neck.

**Table 3:** Blood gas analysis

Parameter	Group	Time point		
		
		T0	T1	T2
SaO_2 _(%)	NTG	99 ± 0	99 ± 0	99 ± 0
	NaCl	99 ± 0	99 ± 0	99 ± 0
ScvO_2 _(%)	NTG	88 ± 0	86 ± 0	89 ± 0
	NaCl	83 ± 0*	85 ± 0	87 ± 0
PaO_2 _(kPa)	NTG	23 ± 2	32 ± 2**	37 ± 5**
	NaCl	23 ± 2	34 ± 3**	36 ± 3**
Hb (mmol/l)	NTG	6.2 ± 0.3	5.6 ± 0.2**	6.0 ± 0.2
	NaCl	6.4 ± 0.7	6.3 ± 0.5*	5.9 ± 0.8**

Values are expressed as mean ± standard error. Hb, haemoglobin; NaCl, normal saline (control); NTG, nitroglycerin; PaO_2_, arterial oxygen tension; SaO_2_, arterial haemoglobin oxygen saturation; ScvO_2_, central venous haemoglobin oxygen saturation; T0, baseline; T1, after construction of the gastric tube; T2, after pulling up the gastric tube to the neck. *P < 0.05 NTG versus NaCl (control); **P < 0.05 versus T0 (baseline).
